# Uniaxial Mechanical Strain Modulates the Differentiation of Neural Crest Stem Cells into Smooth Muscle Lineage on Micropatterned Surfaces

**DOI:** 10.1371/journal.pone.0026029

**Published:** 2011-10-07

**Authors:** Xian Li, Julia Chu, Aijun Wang, Yiqian Zhu, Wai Keung Chu, Li Yang, Song Li

**Affiliations:** 1 Bioengineering College, Chongqing University, Chongqing, China; 2 Department of Bioengineering, University of California, Berkeley, California, United States of America; 3 Department of Bioengineering, University of California San Diego, La Jolla, California, United States of America; University of California, Merced, United States of America

## Abstract

Neural crest stem cells (NCSCs) play an important role in the development and represent a valuable cell source for tissue engineering. However, how mechanical factors *in vivo* regulate NCSC differentiation is not understood. Here NCSCs were derived from induced pluripotent stem cells and used as a model to determine whether vascular mechanical strain modulates the differentiation of NCSCs into smooth muscle (SM) lineage. NCSCs were cultured on micropatterned membranes to mimic the organization of smooth muscle cells (SMCs), and subjected to cyclic uniaxial strain. Mechanical strain enhanced NCSC proliferation and ERK2 phosphorylation. In addition, mechanical strain induced contractile marker calponin-1 within 2 days and slightly induced SM myosin within 5 days. On the other hand, mechanical strain suppressed the differentiation of NCSCs into Schwann cells. The induction of calponin-1 by mechanical strain was inhibited by neural induction medium but further enhanced by TGF-β. For NCSCs pre-treated with TGF-β, mechanical strain induced the gene expression of both calponin-1 and SM myosin. Our results demonstrated that mechanical strain regulates the differentiation of NCSCs in a manner dependent on biochemical factors and the differentiation stage of NCSCs. Understanding the mechanical regulation of NCSC differentiation will shed light on the development and remodeling of vascular tissues, and how transplanted NCSCs respond to mechanical factors.

## Introduction

Human neural crest stem cells (NCSCs) are multipotent stem cells that can be isolated from pluripotent stem cells, the embryonic tissues and the bulge of hair follicles in adult tissue, with relatively low abundance in adult tissues compared to embryo tissues [1], [2], [3]. NCSCs have the potential to differentiate into cell types of all three germ layers and generate a wide variety of cell types during vertebrate development, such as vascular smooth muscle cells (SMCs), Schwann cells, peripheral neurons, osteoblasts, chondrocytes, melanocytes and endocrine cells. In addition, NCSCs can be derived from embryonic stem cells (ESCs and iPSCs) , making them a valuable stem cell source for tissue regeneration and an ideal model system for studying the lineage commitment and therapeutic potential of stem cells. The multipotency of NCSCs can be characterized by their differentiation into neural and mesenchymal lineages. Although there are studies on the regulation of NCSC differentiation by biochemical cues, little is known on whether and how mechanical factors in the microenvironment regulate NCSC differentiation.

Blood vessel wall is constantly subjected to cyclic mechanical strain in the circumferential direction due to the pulsatile nature of the blood flow. It has been reported that mechanical strain plays an important role in the remodeling of blood vessels and tissue-engineered vascular grafts [4], [5], [6], [7], [8]. The application of mechanical strain to SMCs increases the expression of SMC markers, such as smooth muscle myosin heavy chain (MHC) and caldesmon [4], [5], [6], [7], [8], [9], [10], suggesting that mechanical strain may promote SMC contractile phenotype. Recent studies have shown that cyclic mechanical strain increased SMC marker expression in bone marrow mesenchymal stem cells (MSCs) [11], [12], [13]. NCSCs are an important source of vascular SMCs during the vascular development [14], [15]. NCSCs could also be used to construct tissue engineered vascular grafts. Our recent study showed that NCSCs in vascular grafts could differentiate into SMCs *in vivo* (Zhu and Li, unpublished observation). However, whether and how hemodynamic factors regulate NCSC differentiation is unknown. We hypothesized that mechanical strain regulates NCSC differentiation into SMC lineage, and we used iPSC-derived NCSCs as a model to investigate this possibility.

Among biochemical signaling molecules, transforming growth factor β (TGF-β) plays an important role in SMC differentiation. TGF-β regulates gene expression through both Smad-dependent and Smad-independent pathways in a context-dependent manner [16], [17], [18]. TGF-β has been shown to up-regulate the expression of SMC markers in SMCs [19], [20], [21], [22], MSCs [23], [24], [25], ESCs [25] and adult NCSC lines [26]. It's also shown that there is TGF-β-mediated signaling pathways can crosstalk with mechanical force-induced signaling and gene expression in MSCs and endothelial cells [27], [28], [29]. However, whether TGF-β and mechanical strain cooperate to induce SMC differentiation in NCSCs is not clear.

In this study we investigated the effects of mechanical strain on human NCSCs, particularly with regard to the differentiation of NCSCs into smooth muscle lineage. Our results showed that mechanical strain induced contractile markers calponin-1 (CNN1) and MHC, and suppressed the differentiation of NCSCs into Schwann cells. The induction of CNN1 by mechanical strain was inhibited by neural induction medium but further enhanced by TGF-β. For NCSCs pre-treated with TGF-β, mechanical strain induced the expression of both calponin-1 and MHC. Our results demonstrated that mechanical strain regulates the differentiation of NCSCs in a manner dependent on biochemical factors and the differentiation stage of NCSCs. These findings are novel and are important for the understanding of vascular development and how mechanical factors regulate NCSC differentiation *in vivo*.

## Materials and Methods

### Cell culture and NCSC derivation

An iPSC line derived from human bone marrow MSCs (MSC-iPS1) [30] was used to derive NCSCs. To derive NCSCs, iPSCs were detached by collagenase IV (1 mg/ml) and dispase (0.5 mg/ml), and grown as embryo body (EB)-like floating cell aggregates in ESC maintenance medium without basic fibroblast growth factor (bFGF) for 5 days. The cell aggregates were allowed to adhere to the CellStart (A10142-01, Invitrogen Corp., Carlsbad, CA, USA)-coated dishes in StemPro® NSC serum-free neural induction medium (A10509-01, SFM, Invitrogen Corp.). After 7 more days, the colonies with rosette structures were mechanically harvested, cultured in suspension in SFM for 1 week, replanted onto the CellStart-coated dishes, and cultured for 3 more days. Cells were dissociated into single cells by TryplE Select (A1217702, Invitrogen Corp.), cultured in SFM as a monolayer, and further purified by cloning, subculture and/or flow-activated cell sorting (FACS) to obtain homogeneous populations that were positive for neural crest markers. The NCSCs derived from iPSCs were maintained in SFM for expansion without differentiation.

### NCSC differentiation

To demonstrate the multipotency of NCSCs, we carried out the protocol of NCSC differentiation into neural lineages (Schwann cells, peripheral neurons) and mesenchymal lineages (SMCs, adipocytes, osteoblasts and chondrocytes) [2].

For NCSC differentiation into Schwann cells, NCSCs were cultured for 2 weeks in N_2_ medium supplemented with 10 ng/ml ciliary neurotrophic factor (CNTF; RD257-NT, R&D Systems Inc., Minneapolis, MN, USA), 10 ng/ml bFGF (PHG0024, Invitrogen Corp.), 1 mM dibutyryl-cAMP (dbcAMP; D0260, Sigma-Aldrich Corp., St. Louis, MO, USA) and 20 ng/ml neuregulin1β (377-HB, R&D Systems Inc.), and the cells were double-immunostained for Schwann cell markers S100β (S1542, Sigma-Aldrich Corp.) and Glial fibrillary acidic protein (GFAP; AB5804, Millipore Corp., Billerica, MA, USA).

For NCSC differentiation into peripheral neurons, NCSCs were cultured for 2 weeks in N_2_ medium supplemented with 20 ng/ml brain-derived neurotrophic factor (BDNF; 248-BD, R&D Systems Inc.), 10 ng/ml nerve growth factor (NGF; 256-GF, R&D Systems Inc.), 10 ng/ml glial cell line-derived neurotrophic factor (GDNF; 212-GD, R&D Systems Inc.) and 1 mM dbcAMP, and the cells were double-immunostained for peripheral neuron markers peripherin (AB1530, Millipore Corp.) and class-III β-tubulin (TUJ1; MMS-435P, Covance Inc., Princeton, NJ, USA).

For NCSC differentiation into SMC lineage, NCSCs were treated with 10 ng/ml TGF-β1 (100-21, Pepro Tech Inc., Rocky Hill, NJ, USA) in Minimum Essential Medium α Medium (αMEM medium; Invitrogen Corp.) containing 10% Fetal Bovine Serum (FBS; Invitrogen Corp.) for 2 weeks, and double-immunostained with smooth muscle markers CNN1 (1806-1, Epitomics Inc., Burlingame, CA, USA) and smooth muscle α-actin (SMA; A5228, Sigma-Aldrich Corp.).

For adipogenic differentiation, confluent NCSCs were treated with 10 µg/ml insulin (12643, Sigma-Aldrich Corp.), 1 mM dexamethasone (D4902, Sigma-Aldrich Corp.) and 0.5 mM isobutylxanthine (I7018, Sigma-Aldrich Corp.) in αMEM medium containing 10% FBS for 3 weeks. The formation of lipid and fat was examined by phase contrast microscopy and Oil red staining.

For osteogenic differentiation, NCSCs were seeded at a low density (10^3^cells/cm^2^) and grown in αMEM medium containing 10% FBS for 4 weeks in the presence of 10 mM β-glycerol phosphate (Sigma-Aldrich Corp.), 0.1 µM dexamethasone and 200 µM ascorbic acid (Sigma-Aldrich Corp.). Then cells was fixed in 4% paraformaldehyde, and stained with Alizarin red for calcified matrix or immunostained for alkaline phosphatase (ALP; B4-78, DSHB, Iowa city, IA, USA).

For chondrogenic differentiation, NCSC pellets were cultured in suspension in αMEM medium containing 10% FBS for 4 weeks in the presence of 10 ng/ml TGF-β3 (243-B3, R&D Systems Inc.), 0.1 µM dexamethasone and 200 µM ascorbic acid. The cell pellets were cryosectioned, and stained for glycosaminoglycans (GAGs) by using Alcian blue staining or immunostained for Collagen type II (Col-II; MAB8887, Millipore Corp.).

### Microfabrication and soft lithography

Microfabrication techniques were used to create micropatterned elastic membranes with parallel microgrooves (10-µm wide, 3-µm deep, 10-µm spacing between adjacent grooves) [12]. Briefly, photoresist (OIR 897-10I; Arch Chemicals, Norwalk, CT, USA) was spin-coated onto a silicon wafer, and a patterned photomask was used to expose the photoresist to UV light. After washing away the unpolymerized photoresist, polydimethylsiloxane (PDMS) was prepared according to the manufacturer's protocol (Sylgard 184; Dow Corning, Midland, MI, USA), spin-coated onto the patterned silicon wafers to the desired thickness (∼250 µm), degassed under vacuum, and cured at 70°C for 1 hour. The resulting micropatterned membranes were removed from the template, cut to appropriate dimensions for the assembly into custom-built stretch chambers, and thoroughly washed and sonicated before use. The surface topography of micropatterned PDMS membranes was examined by phase contrast microscopy. Mechanical testing showed that PDMS membranes were elastic under cyclic uniaxial strain (<30% strain, 1 Hz).

### Applying cyclic uniaxial strain to NCSCs

For mechanical strain experiments, micropatterned membranes were treated with O_2_ plasma in a Plasma-Prep II plasma etcher (Structure Probe Inc., West Chester, PA, USA) for 1 min, and coated with a 2% gelatin solution. Membranes were assembled into custom-built uniaxial stretch chambers with microgrooves oriented parallel to the axis of strain [12], [31]. Previously we have shown that aligned microgrooves can maintain cell orientation in the direction of mechanical strain [12].

NCSCs were seeded at 80% confluency on the micropatterned membranes in Dulbecco's Modified Eagle's Medium (DMEM; Invitrogen Corp.) supplemented with 10% FBS, 1% penicillin-streptomycin (Invitrogen Corp.) and 1% fungizone (Invitrogen Corp.). For the samples subjected to uniaxial strain, the polysulfone bar at one end of the chamber was attached to a motorized cam/rotor system, while the other end of the frame remained stationary, thereby stretching the membrane [12]. For the no-strain controls, the polysulfone bar still moved back and forth, but the other end of the frame was not held stationary so that the entire membrane moved without being strained but experienced similar fluid disturbance to the stretched membranes in the stretch chamber. NCSCs were subjected to a physiological level of cyclic uniaxial strain at a 5% magnitude and 1 Hz frequency.

### Immunostaining and microscopy

NCSCs were fixed with 4% paraformaldehyde in phosphate-buffered saline (PBS) for 15 min, followed by permeabilization with 0.5% Triton X-100 in PBS for 10 min. For F-actin staining, the specimens were stained with FITC-conjugated phalloidin (A12379, Invitrogen Corp.) for 1 h. For other stainings, the specimens were incubated with respective primary antibodies for 2 h, and with appropriate secondary antibodies for 1 h. Nuclei were stained by DAPI (D1306, Invitrogen Corp.) in blue or propidium iodide (Sigma-Aldrich, Corp.) in red. The following antibodies were used: p75 (AB8874, Abcam Inc., Cambridge, MA, USA), HNK1 (C6680, Sigma-Aldrich Corp.), vimentin (M0725, Dako North America Inc., Carpinteria, CA, USA), nestin (SC-21247, Santa Cruz Biotechnology Inc., Santa Cruz, CA, USA), S100β, GFAP, peripherin, TUJ1, CNN1, SMA, ALP, Col-II and MHC (SC-6956, Santa Cruz Biotechnology Inc.).

The fluorescently stained samples were imaged by using a Leica (Wetzlar, Germany) True Confocal Scanner SL confocal microscopy system, including He/Ne laser sources and a Leica DM IRB microscope, to capture multiple Z-section images (0.3- to 0.5-µm-thick sections) for a given specimen. The optical sections were subsequently projected to a single plane to create an overall image of the specimen. All images in a given group were collected with the same hardware and software settings. Other staining samples were examined using a Zeiss fluorescence microscope. The setting of microscopy system such as exposure time was kept constant for each experiment.

### RNA isolation and quantitative polymerase chain reaction (qPCR)

Cells were lysed with 1 ml of Trizol (15596-026, Invitrogen Corp.) per membrane. RNA was extracted by using chloroform and phenol extractions, precipitated by isopropanol, and the resulting RNA pellet was washed with 75% ethanol. RNA pellets were resuspended in 20 µl DEPC-treated H_2_O and were quantified by RiboGreen® RNA Quantification Reagent and Kit (R11490, Invitrogen Corp.). cDNA was synthesized by using two-step reverse transcription (RT) with the ThermoScript RT-PCR system (11146-024, Invitrogen Corp.), followed by qPCR using SYBR green reagent and the ABI Prism 7000 Sequence Detection System (Life Tech Corp., Carlsbad, CA, USA). Primers for the genes of interest were all designed by using the ABI Prism Primer Express software v.2.0 (Life Tech Corp.) and are listed in Table S1. The gene expression of each sample was normalized to the level of 18S ribosomal RNA of the same sample: 0.5 µg RNA for cDNA synthesis, and 1 µg cDNA for each qPCR reaction. Data was analyzed by using ABI Prism 7000 SDS software (Life Tech Corp.).

### Protein Isolation and immunoblotting

Cells on each membrane were lysed with 100 µl of lysis buffer containing 25 mM Tris (pH 7.4), 0.5 M NaCl, 1% Triton X-100, 0.1% SDS, 1 mM PMSF, 10 µg/ml leupeptin and 1 mM Na_3_VO_4_. Protein lysates were centrifuged to pellet cellular debris, and the supernatant was removed and quantified by DC Protein Assay (Bio-Rad, Hercules, CA). Protein samples (15 µg per well) were run in SDS/PAGE and transferred to nitrocellulose membranes. Membranes were blocked with 3% nonfat milk and incubated with the primary antibodies diluted in TBST (Tris-Buffered Saline Tween-20) buffer containing 25 mM Tris·HCl (pH 7.4), 60 mM NaCl, and 0.05% Tween-20. This was followed by incubation with HRP-conjugated IgG secondary antibodies (Santa Cruz Biotechnology Inc.). To verify the equal loading of proteins in all lanes, nitrocellulose membranes were re-probed with an antibody against actin (SC-1616, Santa Cruz Biotechnology Inc.), including different actin isoforms. Protein bands were visualized by using the Western Lightning Plus-ECL (NEL 10500, Perkin Elmer Life & Analytical Sciences, Shelton, CT, USA), and signal intensity was analyzed by using ImageJ software (NIH). The following primary antibodies were used: p-ERK1/2 (4370s, Cell Signaling Technology, Inc., Danvers, MA, USA), ERK2 (SC-154, Santa Cruz Biotechnologies, Inc.), Tubulin (SC-5286, Santa Cruz Biotechnologies, Inc.), SMA (1184-1, Epitomics Inc.) and CNN1.

### Analysis of cell proliferation

After 1 day of cyclic strain, NCSCs were pulsed with 10 µM BrdU (RPN201, GE Healthcare, Piscataway, NJ, USA) for 2 hours. Then the cells were fixed with 4% paraformaldehyde, permeabilized with 0.5% Triton X-100, and incubated for 30 min in 2N HCl at 37°C. Subsequently, the samples were washed and incubated for 30 min in a buffer containing phosphate buffered saline (PBS), 0.05% Tween-20, and 1 mg/mL BSA to minimize background adsorption of antibodies. The samples were incubated overnight at 4°C with primary antibodies against BrdU (BD347580, BD Biosciences, San Jose, CA, USA) and an FITC-conjugated anti-mouse IgG secondary antibody (715-095-150, Jackson ImmunoResearch Inc., West Grove, PA, USA). After washing with PBS, the stained samples were counterstained with DAPI for 5 min, mounted in Fluoromount G (0100-01, Southern Biotech, Birmingham, AL, USA), and observed with a Zeiss fluorescence microscope. The percentage of NCSCs that incorporated BrdU (i.e., the cells with DNA synthesis) was correlated to the proliferation rate of NCSCs.

### Statistical analysis

Mean and standard deviation (SD) values were calculated for each group of data. Analysis of variance (ANOVA) was performed to detect whether a significant difference existed between groups of different treatments, and a multiple comparison procedure. The *Holm t-test* was used to identify any differences. *Student's t-test* was used to analyze experimental groups with two samples.

## Results

### In vitro characterization of NCSCs

**Figure 1:**
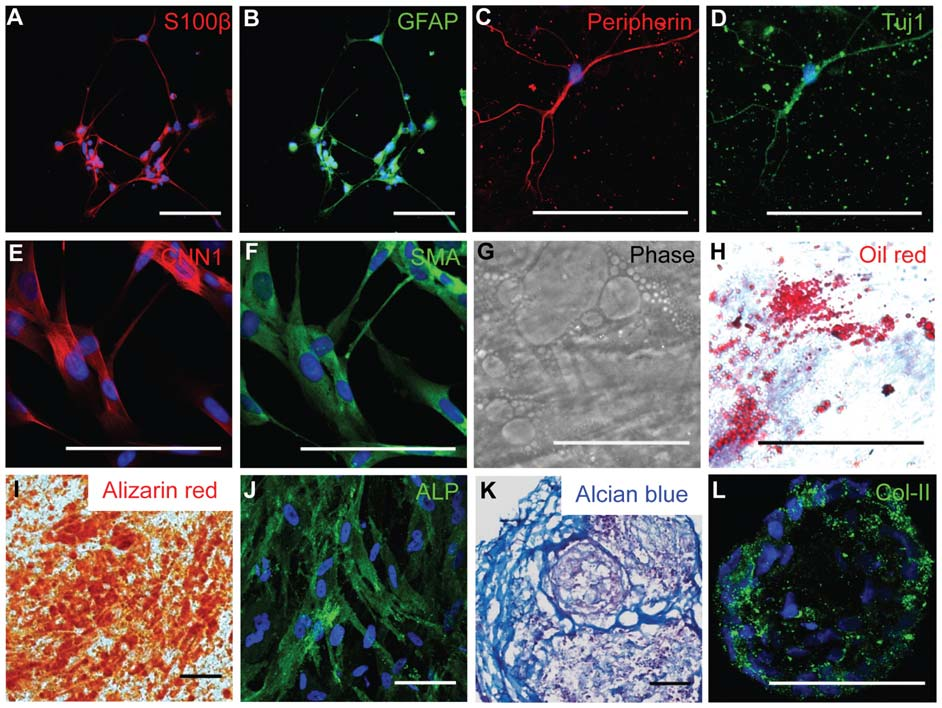
*In vitro* differentiation potential of NCSCs. (A–B) Immunostaining for Schwann cell markers S100β (A) and GFAP (B). (C–D) Immunostaining for peripheral neuron markers peripherin (C) and Tuj1 (D). (E–F) Immunostaining for SMC lineage markers CNN1 (E) and SMA (F). (G–H) Adipogenic differentiation shown by phase contrast imaging (G) and Oil red staining (H). (I–J) Osteogenic differentiation shown by Alizarin red staining for calcified matrix (G) and immunostaining of alkaline phosphatase (ALP) (H). (K–L) Chondrogenic differentiation shown by Alcian blue staining for glycosaminoglycans (E) and immunofluorescent staining of Col-II (F). In all immunofluorescence images, nuclei were stained by DAPI in blue. Scale bar = 100 µm.

Our previous study has shown that NCSCs could be derived from human iPSCs, and NCSCs were positive for neural crest markers (p75, HNK1, vimentin and nestin) and transcriptional factors (slug, AP2) [32]. To verify the multipotency of a specific iPSC-derived NCSC line, we determined whether NCSCs were capable of differentiating into a variety of cell types including neural lineages (Schwann cells, peripheral neurons) and mesenchymal lineages (SMCs, adipocytes and chondrocytes, osteoblasts). As shown in Figure 1, with the treatment of specific differentiation factors, NCSCs differentiated into Schwann cells (Fig. 1A–B), peripheral neurons (Fig. 1C–D), SMC lineage (Fig. 1E–F), adipogenic cells (Fig. 1G–H), osteoblastic cells (Fig. 1I–J), and chondrogenic cells (Fig. 1K–L). These results demonstrated the multipotency of NCSCs.

### Parallel microgrooves maintained NCSC alignment during mechanical strain

**Figure 2:**
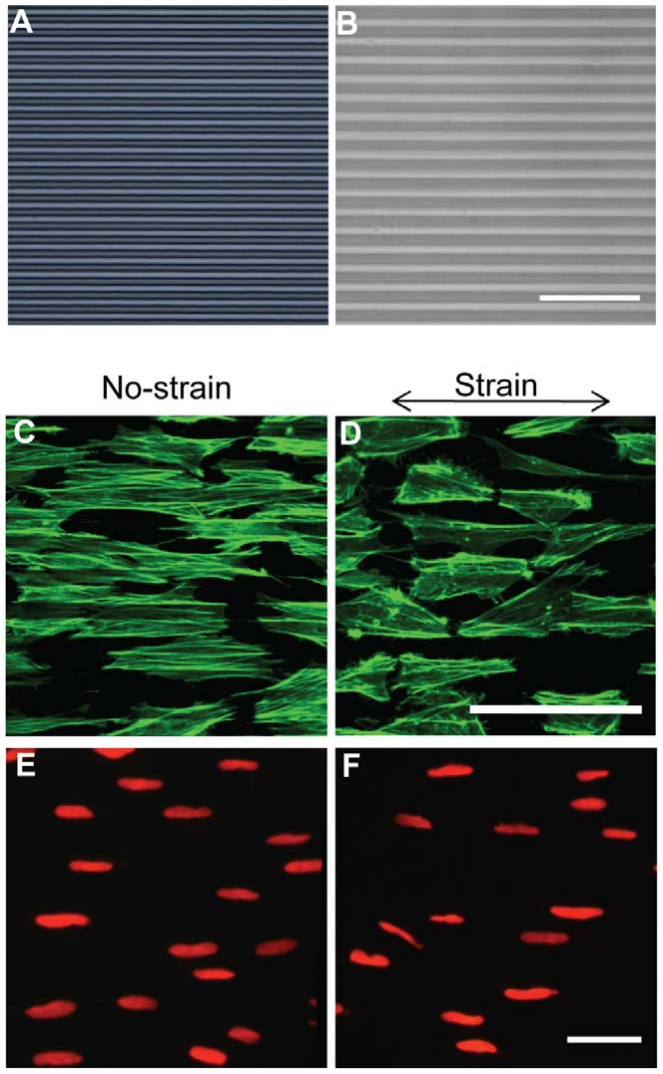
Effects of uniaxial mechanical strain on cell morphology. (A) Micropatterning on the silicon wafer with photoresist. (B) Phase contrast imaging of micropatterned PDMS membranes. NCSCs were cultured on micropattened PDMS membranes and kept as no-strain control (C and E) or subjected to cyclic uniaxial strain (5%, 1 Hz) (D and F) for 1 day. F-actin filaments in NCSCs were stained by phalloidin (C–D). Nuclei were stained by propidium iodide (E–F). Scale bars = 100 µm.

To investigate the effects of mechanical strain on NCSCs, NCSCs were cultured in a general cell culture medium (DMEM with 10% FBS, 1% penicillin-streptomycin and 1% fungizone). NCSCs were subjected to cyclic uniaxial strain within physiological range (5%, 1 Hz). Our previous studies have shown that the direction of cell orientation modulates the effect of mechanical strain on the gene expression in MSCs [12]. To control NCSC orientation, micropatterned elastic PDMS membranes with parallel microgrooves (10 µm wide, 3 µm deep) were fabricated (Fig. 2A–B), and NCSCs were cultured on these micropattened PDMS membranes. NCSCs followed the topographic guidance and aligned with the microgrooves (Fig. 2C). Under uniaxial strain in the direction of cell alignment, NCSCs on parallel-oriented microgrooves remained aligned with the microgrooves and the axis of strain (Fig. 2D). The staining intensity and structure of F-actin did not show significant changes between no-strain and strain conditions. In addition, cell nuclei did not have significant change in morphology and remained aligned in the presence of mechanical strain (Fig. 2E–F).

### Mechanical strain with parallel cell alignment increased NCSC proliferation

**Figure 3:**
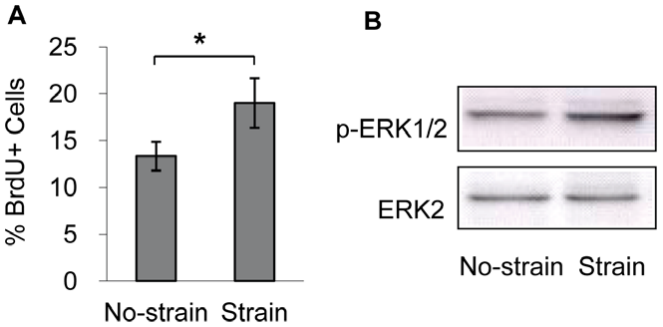
Effects of uniaxial mechanical strain on cell proliferation. NCSCs were cultured on parallel micropatterned surfaces and subjected to cyclic uniaxial strain (5%, 1 Hz) for 1 day. (A) NCSC proliferation. The percentage of BrdU-positive cells (cells in S phase) was calculated. (B) Immunoblotting analysis of p-ERK1/2 and ERK2 after 1 day of cyclic uniaxial strain treatment. * *p*<0.05 in comparison to respective no-strain control using a two-tailed t-test, n≥3.

To investigate the effect of mechanical strain on NCSC proliferation, NCSCs cultured on micropatterned surfaces were subjected to cyclic uniaxial strain for 1 day. Mechanical strain slightly increased the proliferation rate of NCSCs (from 13% to 19%), as indicated by the percentage of NCSCs with BrdU incorporation (Fig. 3A). The activation of ERK1/2 has been shown to play an important role in the proliferation of many cell types. Consistently, mechanical strain increased ERK2 phosphorylation (the lower band in the top panel of Fig. 3B) after 1 day, while the level of ERK1 phosphorylation (the faint band above phosphorylated ERK2) was low. In contrast, mechanical strain had no effect on Akt activation in NCSCs (data not shown).

### Mechanical strain increased the expression of SMC contractile markers and suppressed Schwann cell differentiation

**Figure 4:**
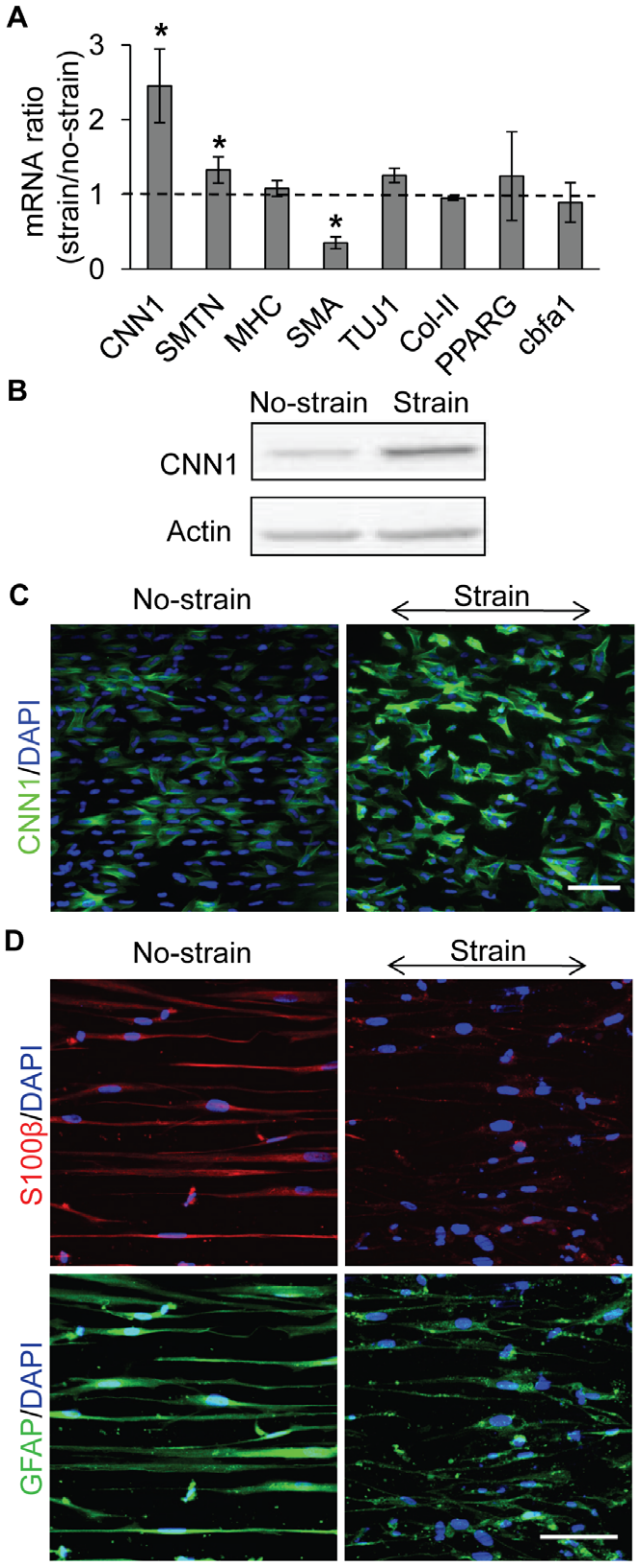
Effects of mechanical strain on gene and protein expression. Cyclic uniaxial strain (5%, 1 Hz, in parallel to microgrooves) was applied to NCSCs for 1 day (for qPCR and immunofluorescent staining) or 2 days (for protein expression analysis). (A) qPCR showing relative gene expression changes after 1 day. CNN1: calponin 1; SMTN: smoothelin; MHC: myosin heavy chain; TUJ1: class-III β-tubulin; Col-II: collagen type II; PPARG: peroxisome proliferator-activated receptor-γ; and cbfa1: core binding factor α-1. * *p*<0.05 in comparison with respective no-strain control by using log-transformed one-sample t-test, n≥3. (B) Immunoblotting analyses showing CNN1 protein expression. The level of total actin was used as equal loading control. (C) Immunofluorescent staining of CNN1. (D) Effect of mechanical strain on NCSC differentiation into Schwann cells. After 2 days' cyclic strain treatment, NCSCs were cultured in Schwann differentiation medium for 1 week, and stained for Schwann cell markers S100β and GFAP. Scale bars = 100 µm.

To determine the effects of mechanical strain on NCSC differentiation, we examined the gene expression of lineage markers including CNN1, MHC, smoothelin (SMTN), TUJ1, Col-II, peroxisome proliferator-activated receptor γ (PPARG), core binding factor α-1 (cbfa1) and vascular endothelial (VE)-cadherin (Fig. 4A). Interestingly, mechanical strain had different effects on SMC contractile markers. Mechanical strain caused a 2.3-fold increase of CNN1 gene expression and a slight increase of SMTN expression, but had no significant effects on the gene expression of MHC. In addition, mechanical strain had no significant effects on neural (TUJ1), chondrogenic (Col-II), adipogenic (PPARG), osteogenic (cbfa1) and endothelial (VE-cadherin expression was not detectable) linage markers.

Using immunoblotting analysis, we found a nearly 70% increase in CNN1 protein expression after 2 days (Fig. 4B). In addition, immunostaining showed that the mechanical strain increased the number of cells (from 20% to 80%) with higher level of CNN1 expression (Fig. 4C) and SMTN (data not shown). These results demonstrated that mechanical strain promoted the differentiation of NCSCs into SMC lineage, but did not induce the expression of mature SMC marker MHC at the early stage (within 2 days).

To determine whether mechanical strain affects the differentiation potential of NCSCs into glial cells, NCSCs were subjected to mechanical strain for 2 days, and cultured in Schwann cell differentiation medium for 1 week. Interestingly, NCSCs under no-strain condition expressed Schwann cell markers S100β and GFAP (Fig. 4D), but pre-stretched NCSCs has little expression of Schwann cell markers. These results suggested that mechanical strain suppressed the differentiation of NCSCs into Schwann cells.

**Figure 5:**
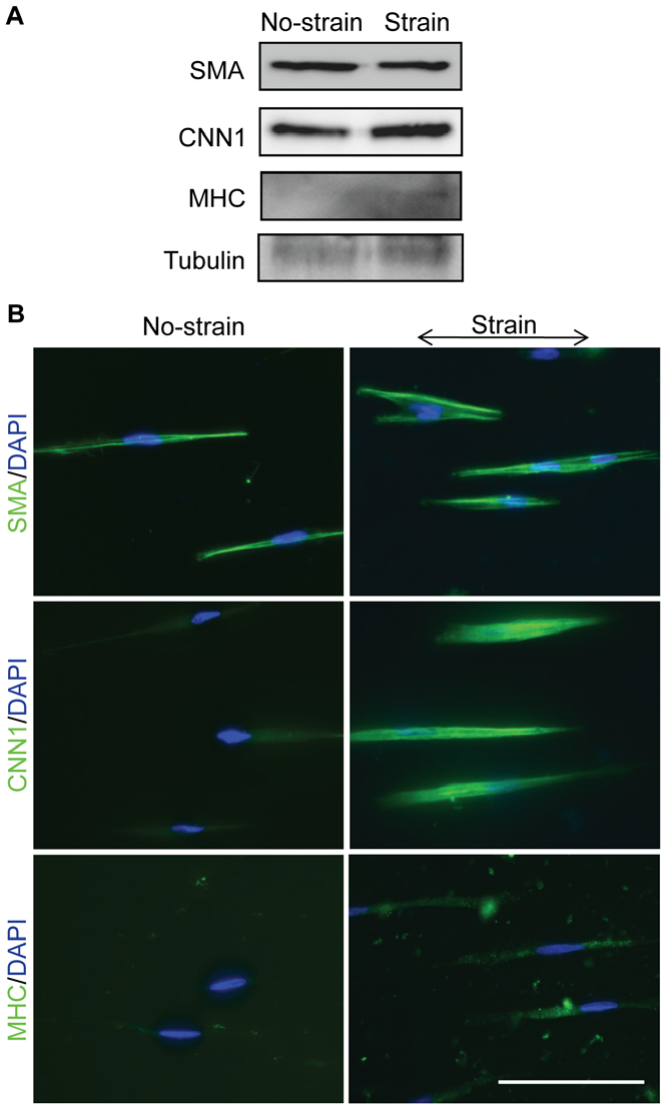
Long-term effects of mechanical strain on NCSC differentiation. Cyclic uniaxial strain (5%, 1 Hz, in parallel to microgrooves) was applied to NCSCs for 5 days. (A) Immunoblotting analysis showing SMA, CNN1 and MHC protein expression. The level of tubulin was used as equal loading control. (B) Immunofluorescent staining of SMA, CNN1 and MHC. Nuclei were stained by DAPI in blue. Scale bar = 100 µm.

Long-term effects of mechanical strain on NCSC differentiation were then investigated. NCSCs were subjected to mechanical strain for 5 days. Immunoblotting analysis showed a 3-fold increase in CNN1 expression, a slight increase in MHC expression, and no significant change in SMA expression (Fig. 5A). Immunostaining experiments further confirmed the changes (Fig. 5B). The slight increase of MHC did not result in the incorporation of MHC into stress fibers, suggesting that the cells had not fully differentiated into SMCs.

### The combined effects of mechanical strain and biochemical factors on NCSC differentiation

**Figure 6:**
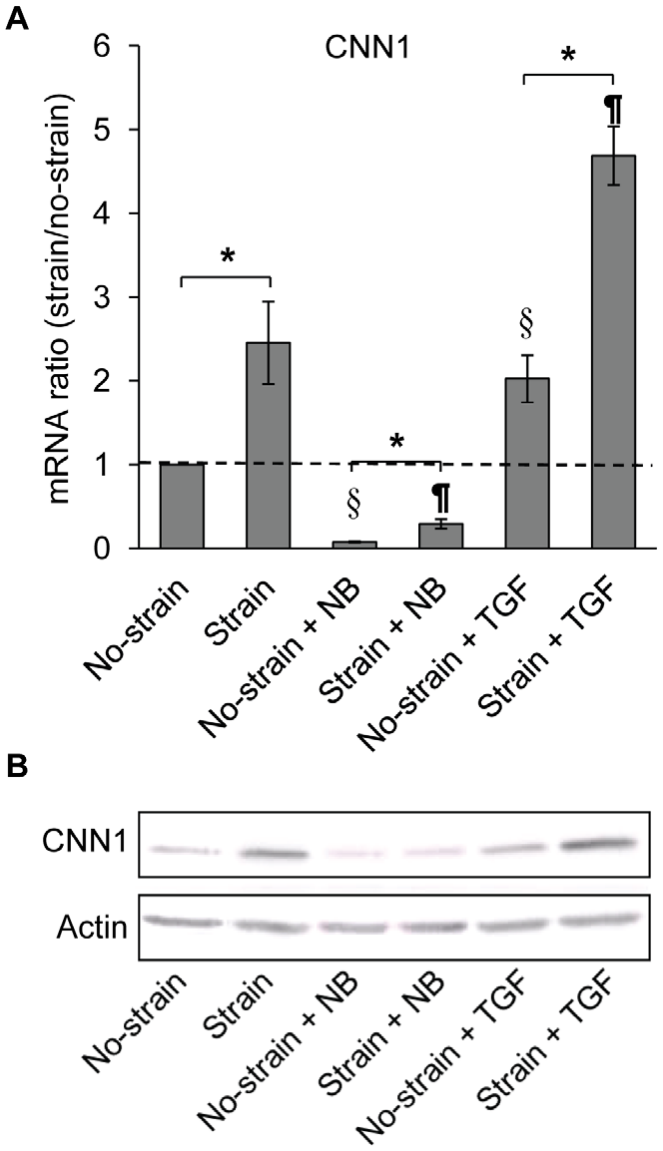
The combined effects of mechanical strain and biochemical factors. NCSCs were cultured in three different types of media: DMEM with10% FBS, neural basal medium (NB) and DMEM with 10% FBS and 10 ng/ml of TGF-β1, and subjected to cyclic strain for 1 day (for qPCR in A) or 2 days (for protein expression analysis in B). * *p*<0.05 in comparison to respective no-strain control by using log-transformed one-sample t-test, n≥3. ^§^ *p*<0.05 in comparison to “no-strain” sample by using log-transformed one-sample t-test, n≥3. ^¶^ *p*<0.05 in comparison to “strain” sample by using log-transformed one-sample t-test, n≥3.

We postulated that different biochemical factors might affect the responses of NCSCs to mechanical strain. To test this possibility, NCSCs were cultured in the general culture medium with or without TGF-β, or cultured in neural basal (NB) medium. NCSCs were subjected to uniaxial strain for 1 day. Biochemical factors had significant effects on the expression level of CNN1. As shown in Figure 6A, NB medium suppressed while TGF-β increased the expression of CNN1 under static condition. However, in all of these culture media, mechanical strain induced CNN1 expression to a similar extent (by fold change), suggesting that mechanical strain regulates CNN1 expression in parallel to the signaling pathways regulated by TGF-β and NB medium. The results of gene expression were verified by CNN1 protein expression (Fig. 6B). In all of three types of culture media, there was no induction of SMC mature marker MHC by mechanical strain within 2 days (data not shown).

### The effect of mechanical strain on NCSC differentiation into SMC lineage at a later stage

**Figure 7:**
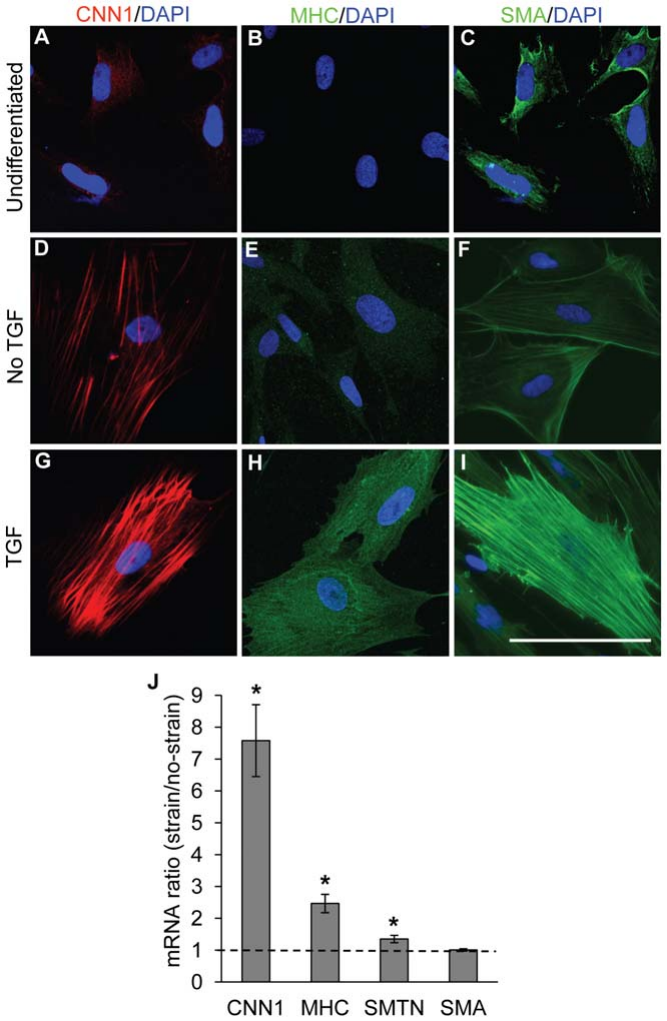
Effect of sequential treatment with TGF-β and mechanical strain. NCSCs were pre-treated with DMEM supplemented with10% FBS and 10 ng/ml of TGF-β1 for 2 weeks, and then subjected to cyclic uniaxial strain for 2 days. (A–I) Immunostaining of undifferentiated NCSCs and NCSCs with or without TGF-β1 treatment for 2 weeks. Scale bar = 100 µm. (J) Gene expression was analyzed by qPCR analysis. * *p*<0.05 in comparison to respective no-strain control by using log-transformed one-sample t-test, n≥3.

To test whether mechanical strain regulated NCSC differentiation at a later stage, we treated NCSCs with TGF-β for 2 weeks, and then subjected the cells to cyclic uniaxial strain for 2 days. Long-term culture of NCSCs in the general culture medium without the supplement of TGF-β resulted in spontaneous differentiation and the increase of SMA, CNN1 and MHC (Fig. 7 A–F) and the assembly of SMA and CNN1 into stress fibers. This “default” differentiation of NCSCs might be resulted from the low level of various growth factors in FBS, e.g., 500–800 pg/ml of TGF-β in FBS. TGF-β treatment further induced the expression of SMA, CNN1 and MHC (Fig. 7 G–I). Mechanical strain significantly increased the gene expression of CNN1 (7.5-fold), MHC (2.5-fold) and SMTN (a slight increase) (Fig. 7 J) after 2 days. These results demonstrated that mechanical strain increased the expression of mature SMC marker (MHC) at a later stage of NCSC differentiation.

## Discussion

It has been shown that micropatterning techniques could be used to control cell morphology to better mimic cell shape and organization in vivo and to investigate anisotropic mechanosensing in stem cells and other cell types [12]. Here we show that uniaxial strain promotes the proliferation and differentiation of NCSCs on micropatterned surfaces. The underlying mechanisms are not clear, but it is likely that mechanical strain activates multiple signaling pathways that resulted in changes in different aspects of cell functions. For example, mechanical strain increased the proliferation and ERK phosphorylation but did not cause Akt activation. However, mechanical strain could also regulate the activity of other mitogen-activated protein kinases such as p38 and JNK, thus modulating cell proliferation. More in-depth studies are needed to dissect the mechanisms of how mechanical strain regulates the proliferation and differentiation of NCSCs. The fact that mechanical strain promotes NCSC differentiation into SMC lineage provides an explanation of how NCSCs differentiate into mature SMC *in vivo*, and suggests that undifferentiated NCSCs could be directly used for vascular tissue engineering.

Previous studies have demonstrated the roles of mechanical stimulation in promoting MSC differentiation into SMC lineage [16], [17], [18], [33]. Several studies have shown that mechanical strain also induces the up-regulation of smooth muscle markers such as CNN1, SMA, and MHC in SMCs [34], [35]. Here we showed that mechanical strain significantly induced the expression of CNN1 (within 2 days) and MHC (after 5 days). However, there was no incorporation of mature SMC marker MHC into stress fibers, suggesting that mechanical strain alone was not sufficient to induce the terminal differentiation of SMCs and that other factors, specifically biochemical factors, are required for the terminal differentiation. In contrast, mechanical strain did not induce the markers of several other lineages, including neural, chondrogenic, adipogenic, osteogenic and endothelial lineages, indicating that mechanical strain has specific effect on NCSC differentiation into SMC lineage. Although mechanical strain did not promote the terminal differentiation of NCSCs, it did affect the differentiation potential of NCSCs into neural linage such as Schwann cells, implicating that mechanical strain could drive NCSC differentiation into an intermediate stage at which the cells partially loss the differentiation potential into other lineages.

Previous studies have shown that TGF-β induces the expression of various smooth muscle markers in MSCs, and the combination of mechanical strain and TGF-β further promoted MSC differentiation into SMCs [19], [22], [23], [24], [25], [29]. In addition, TGF-β induced differentiation of smooth muscle lineage from a mouse NCSC line [26]. Here we showed that both mechanical strain and TGF-β, but no PDGF (data not shown), induced an increase of contractile markers such as CNN1, and the combination of the two factors had additive effects. Even though NB medium suppressed the basal level of SMC marker expression, mechanical strain still induced CNN expression. These results suggest that mechanical strain works in parallel with biochemical factors to regulate NCSC differentiation.

How mechanical strain regulates stem cell differentiation at different stages has not been addressed previously. Here we showed that mechanical strain did not promote MHC expression at the early stage of differentiation but induced MHC expression at the late stage of NCSC differentiation, either after 5 days or following TGF-β treatment. These results suggest that the effects of mechanical strain are dependent on the differentiation stage of NCSCs. However, mechanical strain did not induce SMA expression, suggesting that the signaling mechanisms involved in the expression of SMA and CNN1/MHC in NCSCs may be different.

Overall, our results suggest that mechanical strain plays an important role in the differentiation of NCSCs into smooth muscle lineage. To drive the terminal SMC differentiation, biochemical factors may be needed, and the timing and combination of mechanical and biochemical signaling may be critical. In addition, the differentiation efficiency of NCSCs in response to mechanical strain, in comparison with the cytokine milieu, cellular microenvironment, and conditional activation of specific gene expression, awaits further studies. The underlying mechanisms of mechanotransduction also need to be elucidated.

## Supporting Information

Table S1 **Primers for qPCR.**
(PDF)

## References

[1] Stemple DL, Anderson DJ (1992). Isolation of a stem cell for neurons and glia from the mammalian neural crest.. Cell.

[2] Lee G, Kim H, Elkabetz Y, Al Shamy G, Panagiotakos G (2007). Isolation and directed differentiation of neural crest stem cells derived from human embryonic stem cells.. Nat Biotechnol.

[3] Crane JF, Trainor PA (2006). Neural crest stem and progenitor cells.. Annu Rev Cell Dev Biol.

[4] Seliktar D, Black RA, Vito RP, Nerem RM (2000). Dynamic mechanical conditioning of collagen-gel blood vessel constructs induces remodeling in vitro.. Ann Biomed Eng.

[5] Niklason LE, Gao J, Abbott WM, Hirschi KK, Houser S (1999). Functional arteries grown in vitro.. Science.

[6] Li C, Xu Q (2000). Mechanical stress-initiated signal transductions in vascular smooth muscle cells.. Cell Signal.

[7] Williams B (1998). Mechanical influences on vascular smooth muscle cell function.. J Hypertens.

[8] Kim BS, Nikolovski J, Bonadio J, Mooney DJ (1999). Cyclic mechanical strain regulates the development of engineered smooth muscle tissue.. Nat Biotechnol.

[9] Reusch P, Wagdy H, Reusch R, Wilson E, Ives HE (1996). Mechanical strain increases smooth muscle and decreases nonmuscle myosin expression in rat vascular smooth muscle cells.. Circ Res.

[10] Birukov KG, Shirinsky VP, Stepanova OV, Tkachuk VA, Hahn AW (1995). Stretch affects phenotype and proliferation of vascular smooth muscle cells.. Mol Cell Biochem.

[11] Park JS, Chu JS, Cheng C, Chen F, Chen D (2004). Differential effects of equiaxial and uniaxial strain on mesenchymal stem cells.. Biotechnol Bioeng.

[12] Kurpinski K, Chu J, Hashi C, Li S (2006). Anisotropic mechanosensing by mesenchymal stem cells.. Proc Natl Acad Sci U S A.

[13] Hamilton DW, Maul TM, Vorp DA (2004). Characterization of the response of bone marrow-derived progenitor cells to cyclic strain: implications for vascular tissue-engineering applications.. Tissue Eng.

[14] Hirschi KK, Majesky MW (2004). Smooth muscle stem cells.. Anat Rec A Discov Mol Cell Evol Biol.

[15] Majesky MW (2007). Developmental basis of vascular smooth muscle diversity.. Arteriosclerosis Thrombosis and Vascular Biology.

[16] Topper JN (2000). TGF-β in the cardiovascular system: molecular mechanisms of a context-specific growth factor.. Trends Cardiovasc Med.

[17] Moustakas A, Souchelnytskyi S, Heldin CH (2001). Smad regulation in TGF-β signal transduction.. J Cell Sci.

[18] Derynck R, Akhurst RJ, Balmain A (2001). TGF-β signaling in tumor suppression and cancer progression.. Nat Genet.

[19] Hautmann MB, Madsen CS, Owens GK (1997). A transforming growth factor β (TGFβ) control element drives TGFβ-induced stimulation of smooth muscle α-actin gene expression in concert with two CArG elements.. J Biol Chem.

[20] Adam PJ, Regan CP, Hautmann MB, Owens GK (2000). Positive- and negative-acting Kruppel-like transcription factors bind a transforming growth factor β control element required for expression of the smooth muscle cell differentiation marker SM22α in vivo.. J Biol Chem.

[21] Liu Y, Sinha S, Owens GK (2003). A TGF-β control element required for SM α-actin expression in vivo also partially mediates GKLF-dependent transcriptional repression.. J Biol Chem.

[22] Hirschi KK, Lai L, Belaguli NS, Dean DA, Schwartz RJ (2002). Transforming growth factor-β induction of smooth muscle cell phenotpye requires transcriptional and post-transcriptional control of serum response factor.. J Biol Chem.

[23] Kinner B, Zaleskas JM, Spector M (2002). Regulation of smooth muscle actin expression and contraction in adult human mesenchymal stem cells.. Exp Cell Res.

[24] Wang D, Park JS, Chu JS, Krakowski A, Luo K (2004). Proteomic profiling of bone marrow mesenchymal stem cells upon transforming growth factor β1 stimulation.. J Biol Chem.

[25] Kurpinski K, Lam H, Chu J, Wang A, Kim A (2010). Transforming growth factor-β and notch signaling mediate stem cell differentiation into smooth muscle cells.. Stem Cells.

[26] Chen S, Lechleider RJ (2004). Transforming growth factor-β-induced differentiation of smooth muscle from a neural crest stem cell line.. Circ Res.

[27] Stegemann JP, Nerem RM (2003). Phenotype modulation in vascular tissue engineering using biochemical and mechanical stimulation.. Ann Biomed Eng.

[28] Topper JN, Cai J, Qiu Y, Anderson KR, Xu YY (1997). Vascular MADs: two novel MAD-related genes selectively inducible by flow in human vascular endothelium.. Proc Natl Acad Sci U S A.

[29] Kurpinski K, Chu J, Wang D, Li S (2009). Proteomic Profiling of Mesenchymal Stem Cell Responses to Mechanical Strain and TGF-β1.. Cell Mol Bioeng.

[30] Park IH, Zhao R, West JA, Yabuuchi A, Huo H (2008). Reprogramming of human somatic cells to pluripotency with defined factors.. Nature.

[31] Huang NF, Patel S, Thakar RG, Wu J, Hsiao BS (2006). Myotube assembly on nanofibrous and micropatterned polymers.. Nano Lett.

[32] Wang AJ, Tang ZY, Park IH, Zhu YQ, Patel S (2011). Induced pluripotent stem cells for neural tissue engineering.. Biomaterials.

[33] Gong Z, Niklason LE (2008). Small-diameter human vessel wall engineered from bone marrow-derived mesenchymal stem cells (hMSCs).. FASEB J.

[34] Li S, Fan YS, Chow LH, Van Den Diepstraten C, van Der Veer E (2001). Innate diversity of adult human arterial smooth muscle cells: cloning of distinct subtypes from the internal thoracic artery.. Circ Res.

[35] Cevallos M, Riha GM, Wang X, Yang H, Yan S (2006). Cyclic strain induces expression of specific smooth muscle cell markers in human endothelial cells.. Differentiation.

