# Supplementary material for: Uniaxial Mechanical Strain Modulates the Differentiation of Neural Crest Stem Cells into Smooth Muscle Lineage on Micropatterned Surfaces
Source: PLoS One. 2011 Oct 7;6(10):e26029. doi: 10.1371/journal.pone.0026029 (PMC3189240; doi:10.1371/journal.pone.0026029)
Supplement: Table S1 — Primers for qPCR. (PDF) [file pone.0026029.s001.pdf]

Table 1. Primers for qPCR

Gene name	Forward primer (5' to 3')	Reverse primer (5' to 3')
CNN1	GCATGTCCTCTGCTCACTTCAA	GGGCCAGCTTGTTCTTAACCT
cbfa1	CCAGAAGGCACAGACAGAAGCT	AGGAATGCGCCCTAAATCACT
c-Myc	GGACCCGCTTCTCTGAAAGG	GAGGCTGCTGGTTTTCCACTA
Col-II	GGAAGAGTGGAGACTACTGGATTGAC	TCCATGTTGCAGAAAACCTTCA
Klf4	TCCTTCCTGCCCGATCAG	GGCATGAGCTCTTGGTAATGG
MHC	GCCTCCGTGCTACACAACCT	GCAGAAGAGGCCAGAGTACGTATATA
Nanog	TGCCTCACACGGAGACTGTC	TGCTATTCTTCGGCCAGTTG
Oct3/4	GAGAACAATGAGAACCTTCAGGAGA	TTCTGGCGCCGGTTACAGAACCA
PPARG	TTGAATGTCGTGTCTGTGGAGAT	GGAAGAAACCCTTGCATCCTT
SMA	CAGCTCCAGCTATGTGTGAAGAA	GCAAAGCCGGCCTTACAGA
SMTN	CAGCTGGAGTCCATGAACGA	GCTTCGCAACAGTGCAGTCA
Sox2	CACTGCCCCTCTCACACATG	CCCATTTCCCTCGTTTTTCTT
TUJ1	GGGCCAAGTTCTGGGAAGTC	CGAGTCGCCCACGTAGTTG
18S rRNA	CGCAGCTAGGAATAATGGAATAGG	CATGGCCTCAGTTCCGAAA
